# Supplementary figures and images for: Addressing incomplete lineage sorting and paralogy in the inference of uncertain salmonid phylogenetic relationships
Source: PeerJ. 2020 Jul 3;8:e9389. doi: 10.7717/peerj.9389 (PMC7337038; doi:10.7717/peerj.9389)

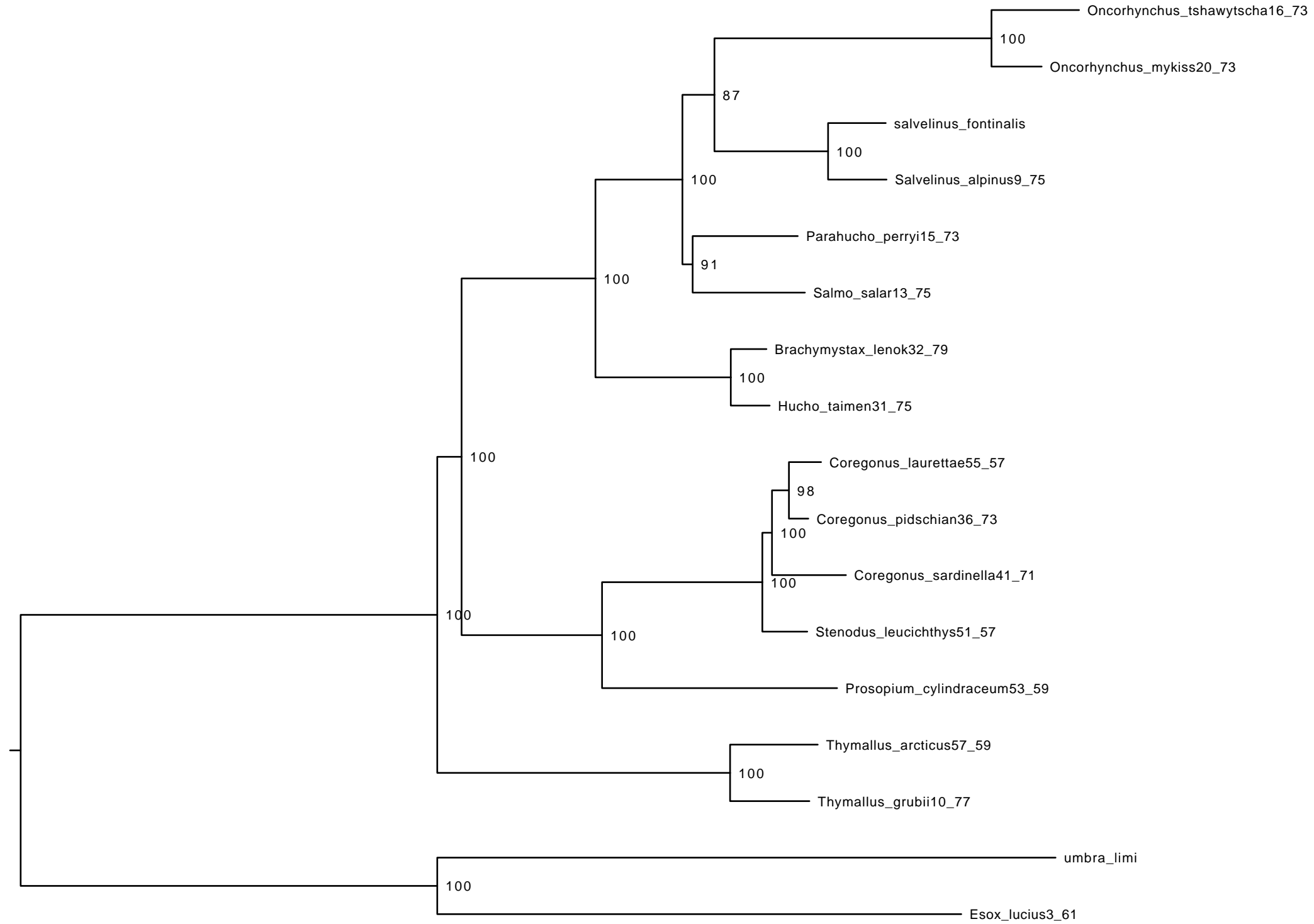

0.01

Supplement: Supplemental Information 1 — Assembled contigs matching UCE loci, alignments, raw code and tree files. [file peerj-08-9389-s001.zip › Data Supplement/RAxML trees/100ByUCE.pdf]

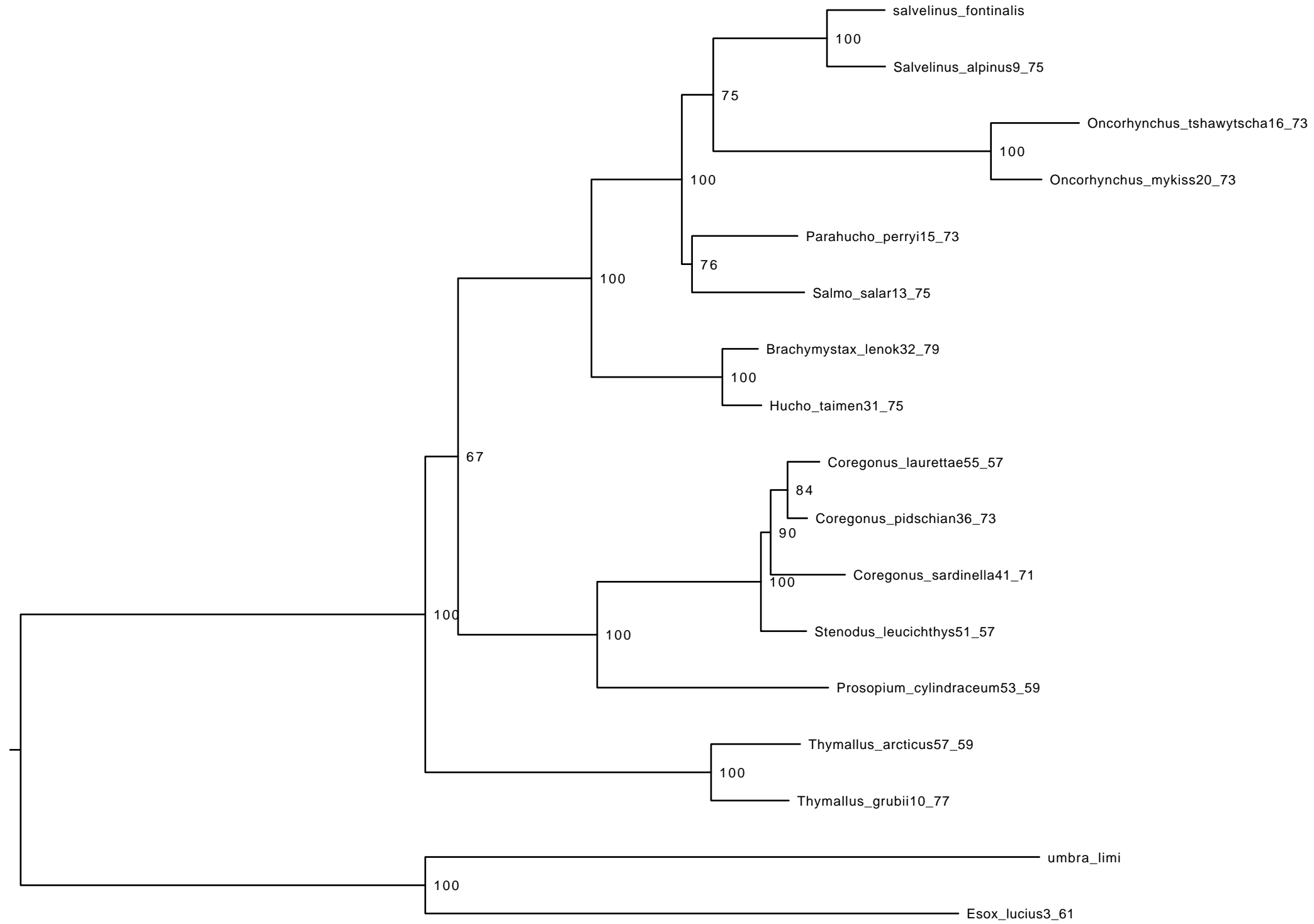

0.01

Supplement: Supplemental Information 1 — Assembled contigs matching UCE loci, alignments, raw code and tree files. [file peerj-08-9389-s001.zip › Data Supplement/RAxML trees/100Unpartitioned.pdf]

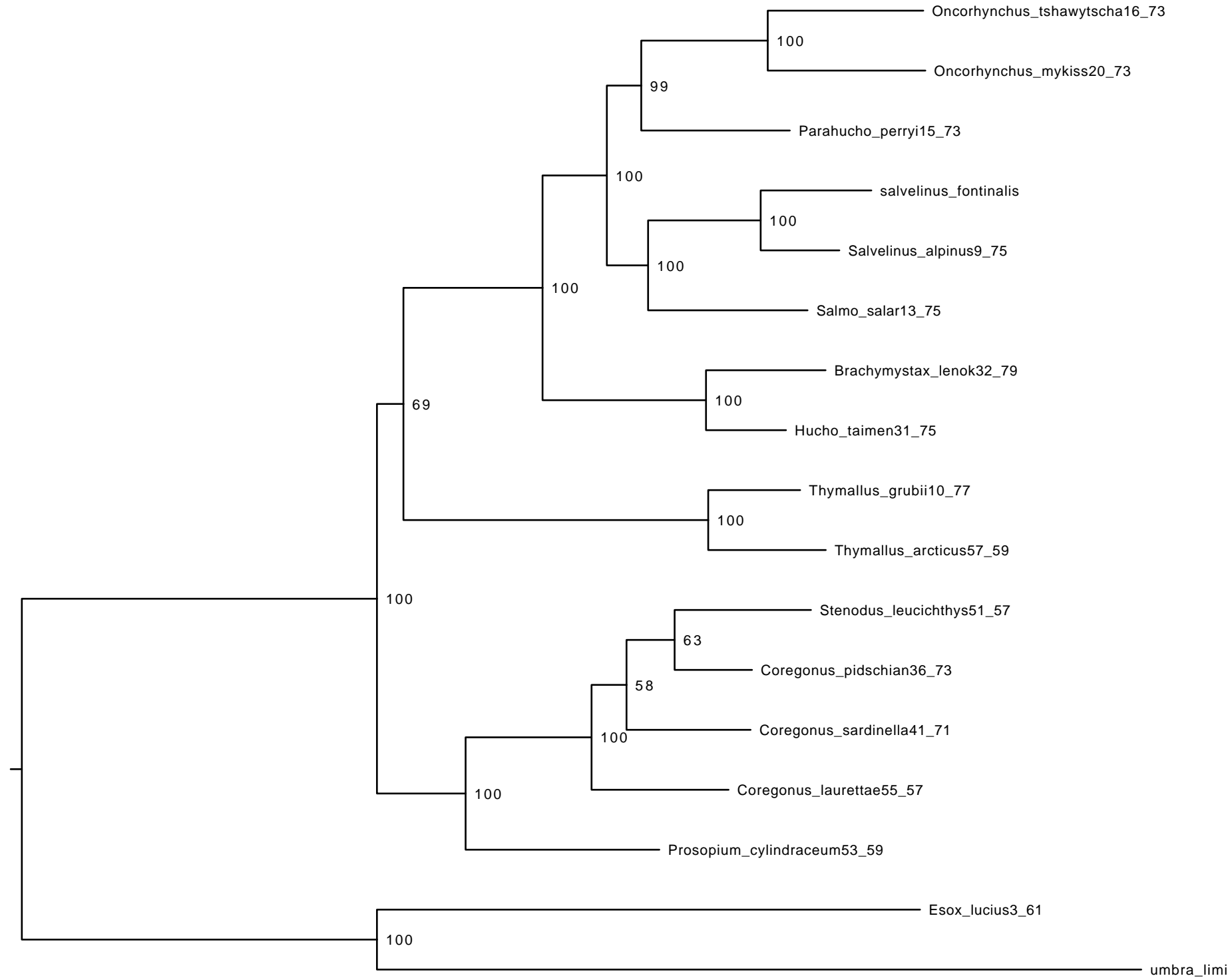

Supplement: Supplemental Information 1 — Assembled contigs matching UCE loci, alignments, raw code and tree files. [file peerj-08-9389-s001.zip › Data Supplement/RAxML trees/75ByUCE.pdf]

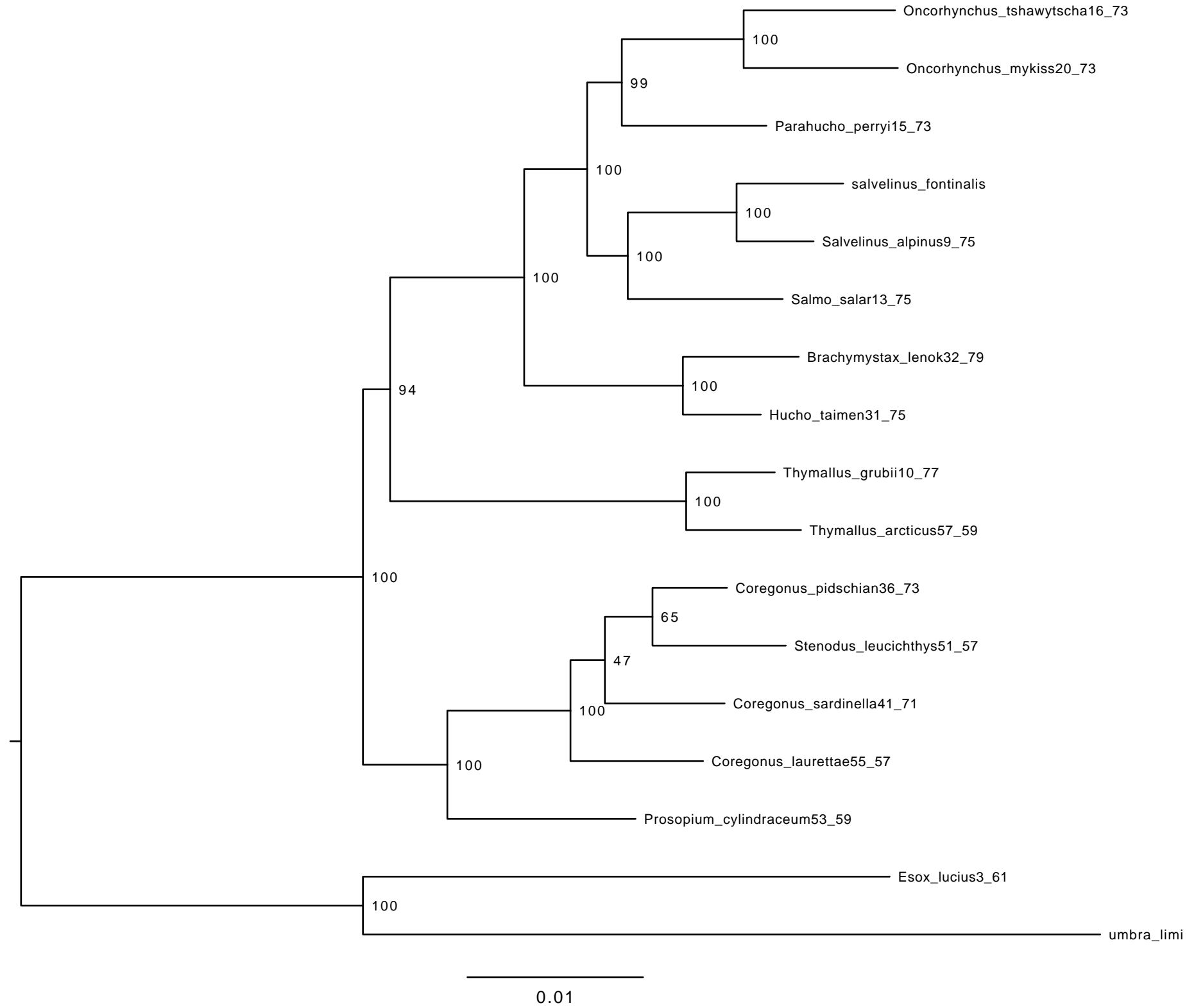

Supplement: Supplemental Information 1 — Assembled contigs matching UCE loci, alignments, raw code and tree files. [file peerj-08-9389-s001.zip › Data Supplement/RAxML trees/75Greedy.pdf]

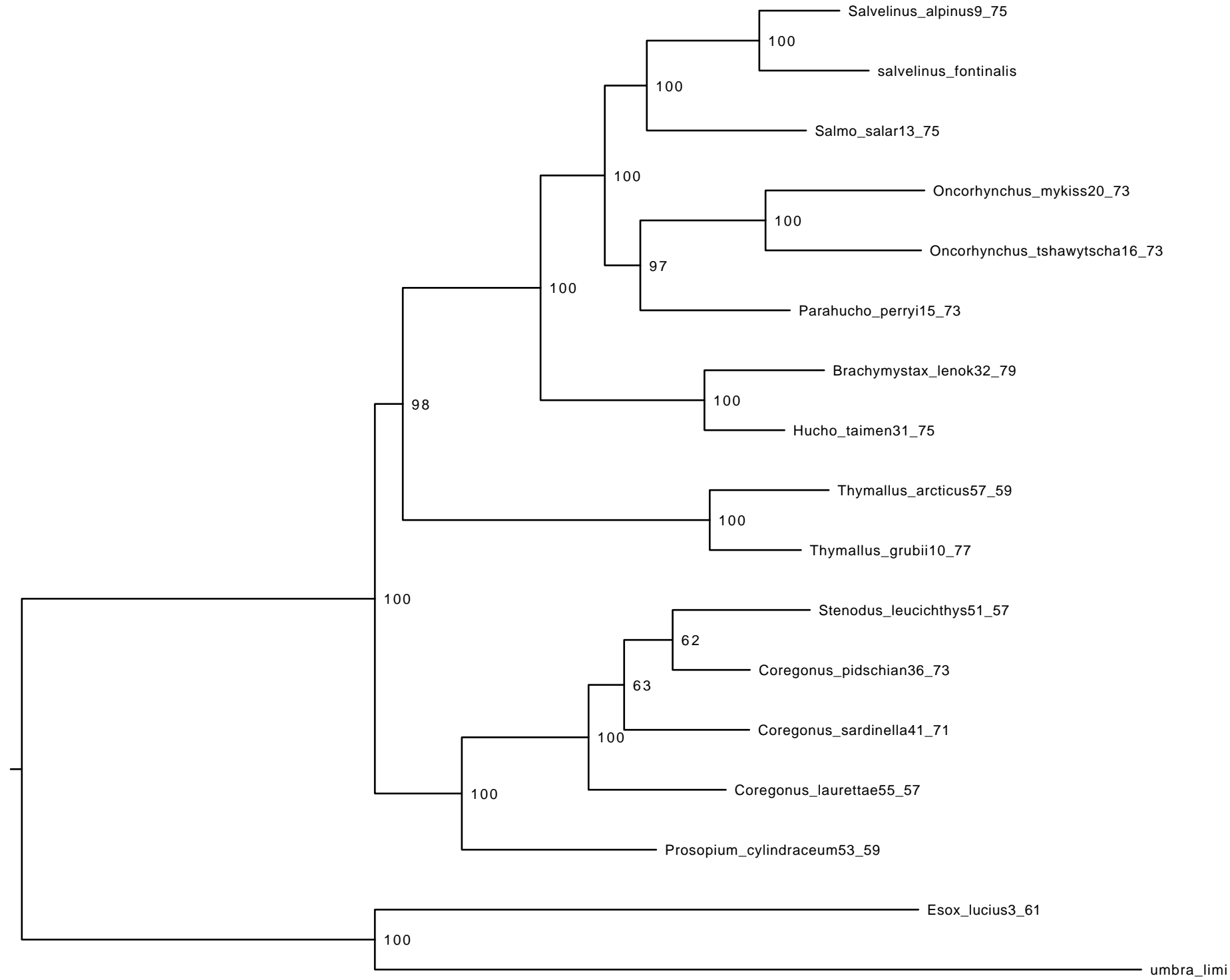

0.01

Supplement: Supplemental Information 1 — Assembled contigs matching UCE loci, alignments, raw code and tree files. [file peerj-08-9389-s001.zip › Data Supplement/RAxML trees/75Unpartitioned.pdf]

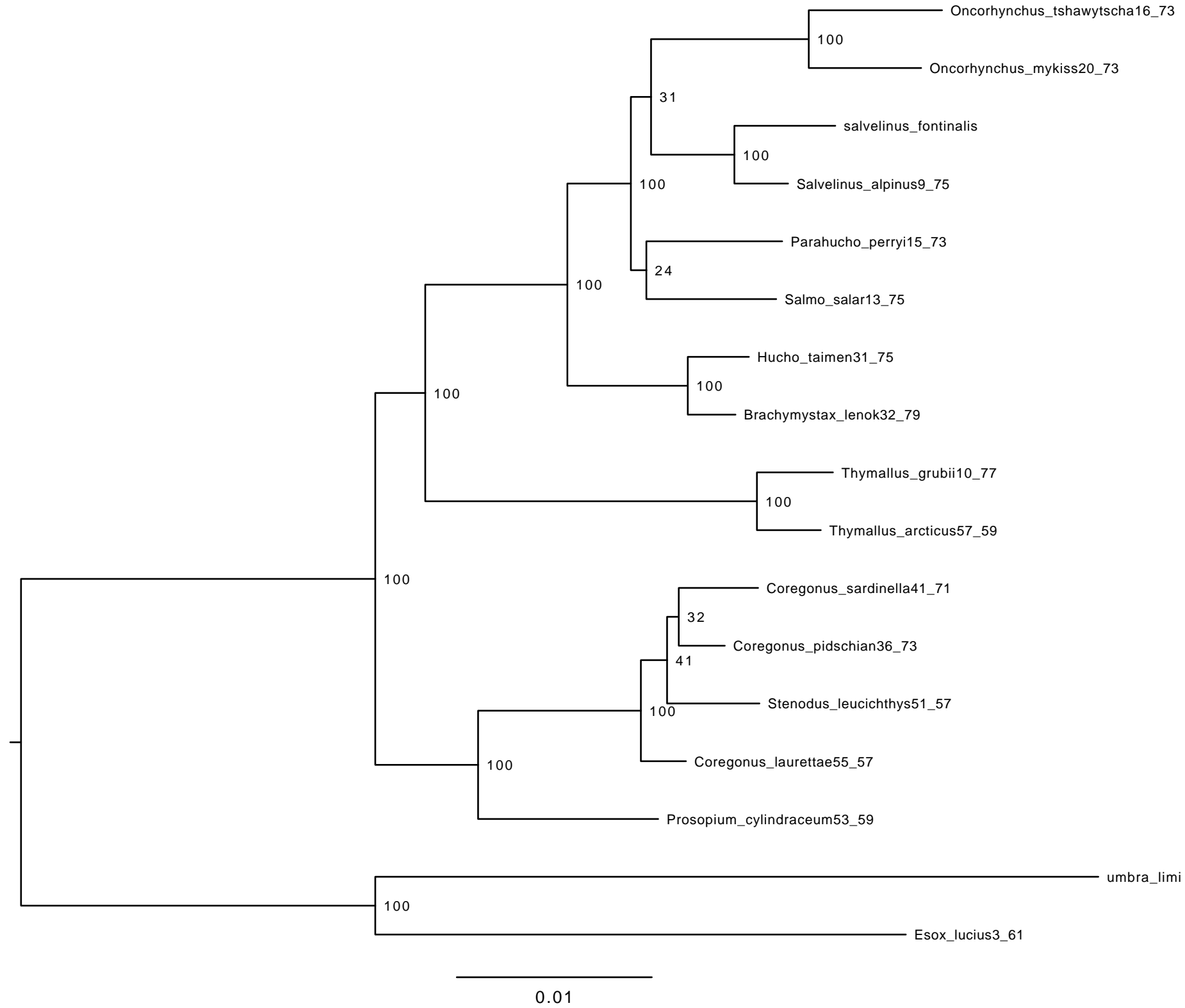

Supplement: Supplemental Information 1 — Assembled contigs matching UCE loci, alignments, raw code and tree files. [file peerj-08-9389-s001.zip › Data Supplement/RAxML trees/93ByUCE.pdf]

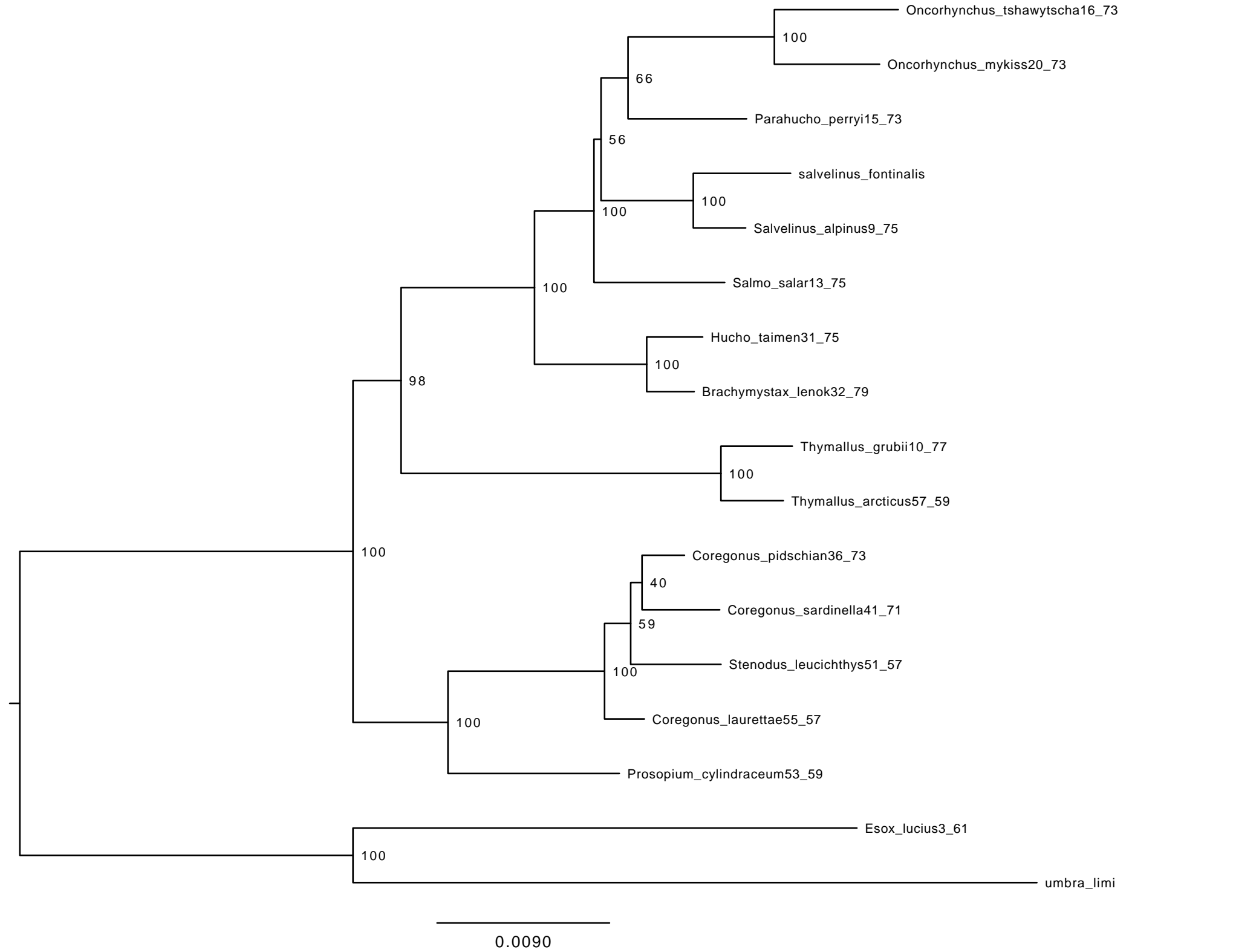

Supplement: Supplemental Information 1 — Assembled contigs matching UCE loci, alignments, raw code and tree files. [file peerj-08-9389-s001.zip › Data Supplement/RAxML trees/93Greedy.pdf]

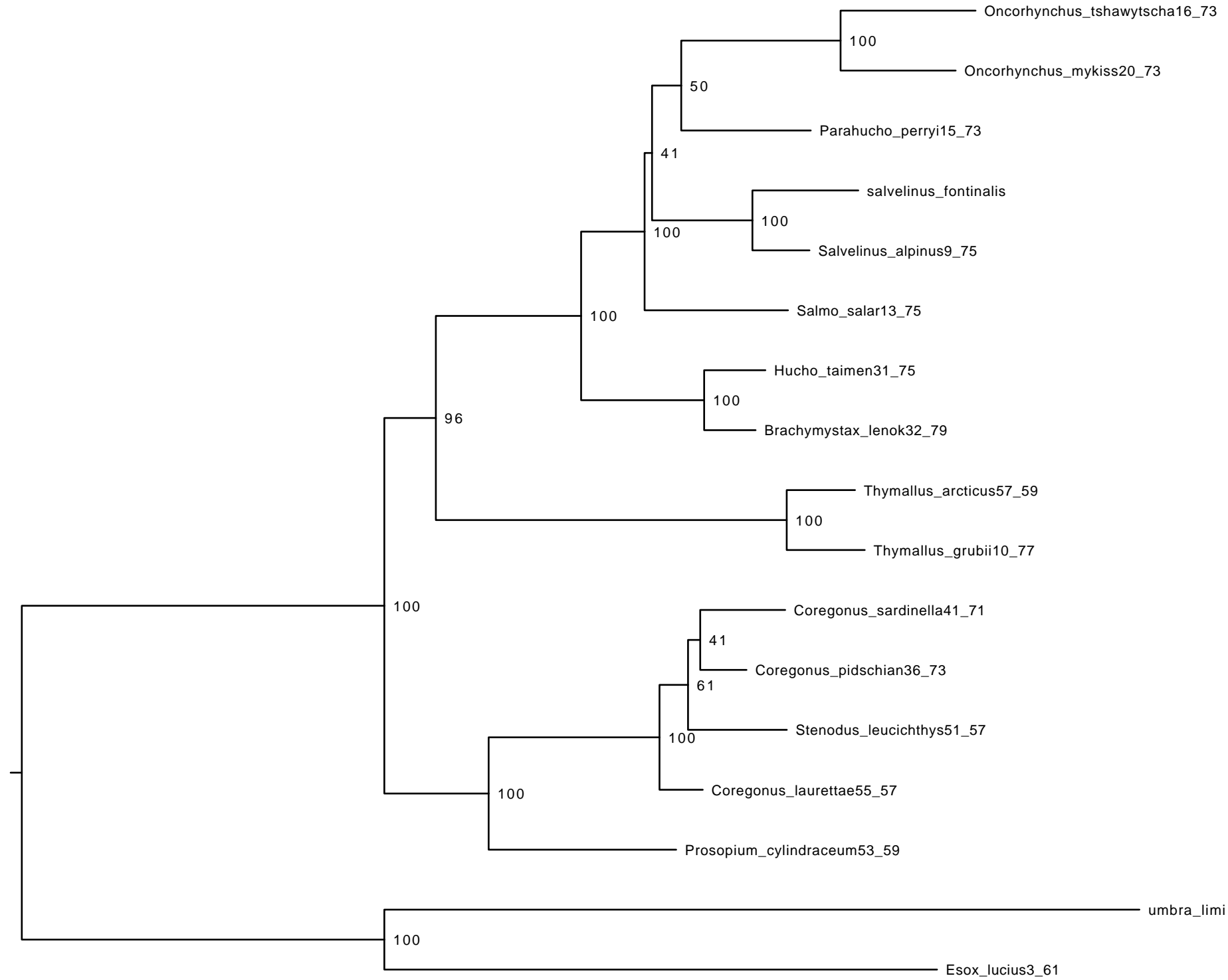

0.0090

Supplement: Supplemental Information 1 — Assembled contigs matching UCE loci, alignments, raw code and tree files. [file peerj-08-9389-s001.zip › Data Supplement/RAxML trees/93Unpartitioned.pdf]

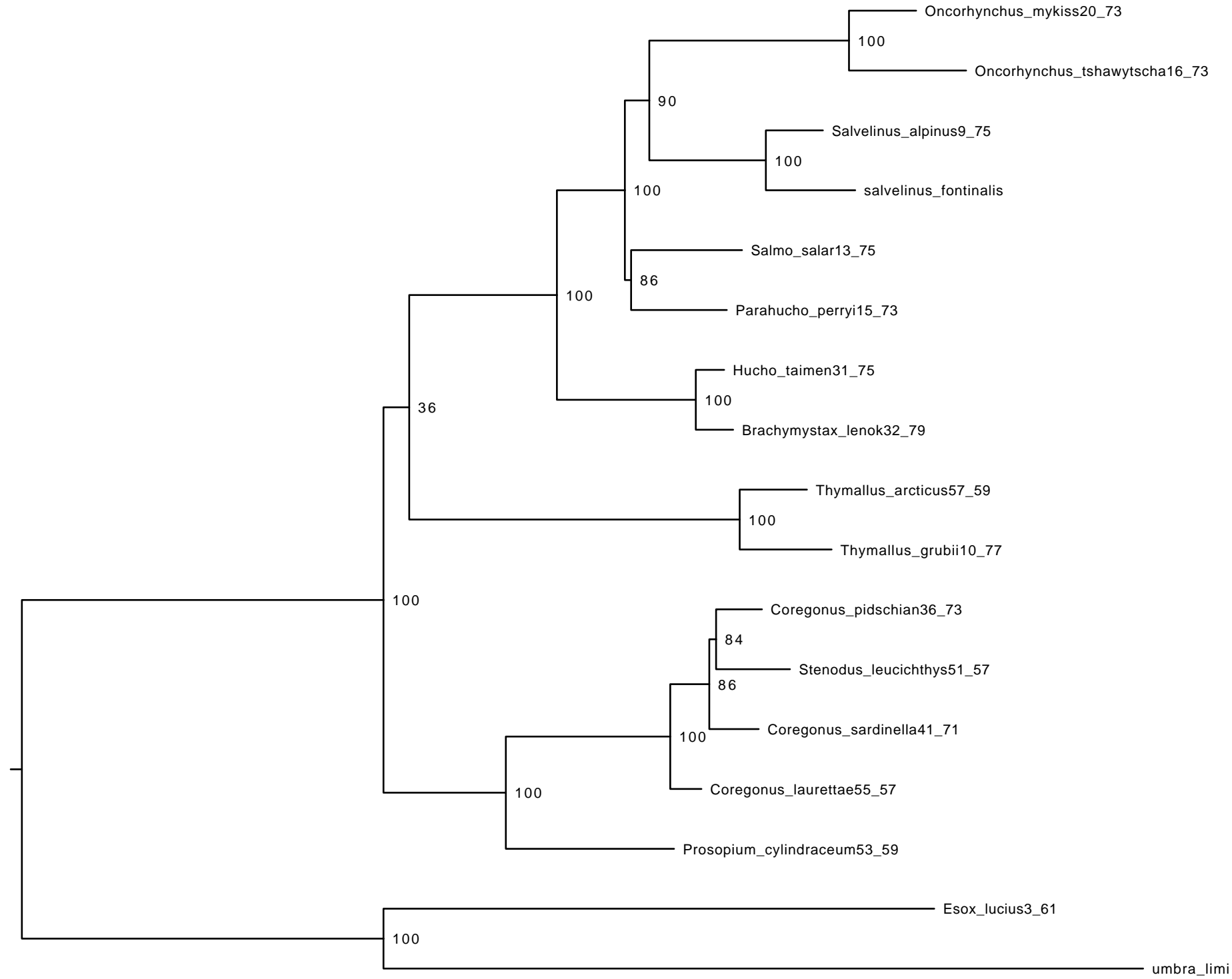

0.01

Supplement: Supplemental Information 1 — Assembled contigs matching UCE loci, alignments, raw code and tree files. [file peerj-08-9389-s001.zip › Data Supplement/RAxML trees/95ByUCE.pdf]

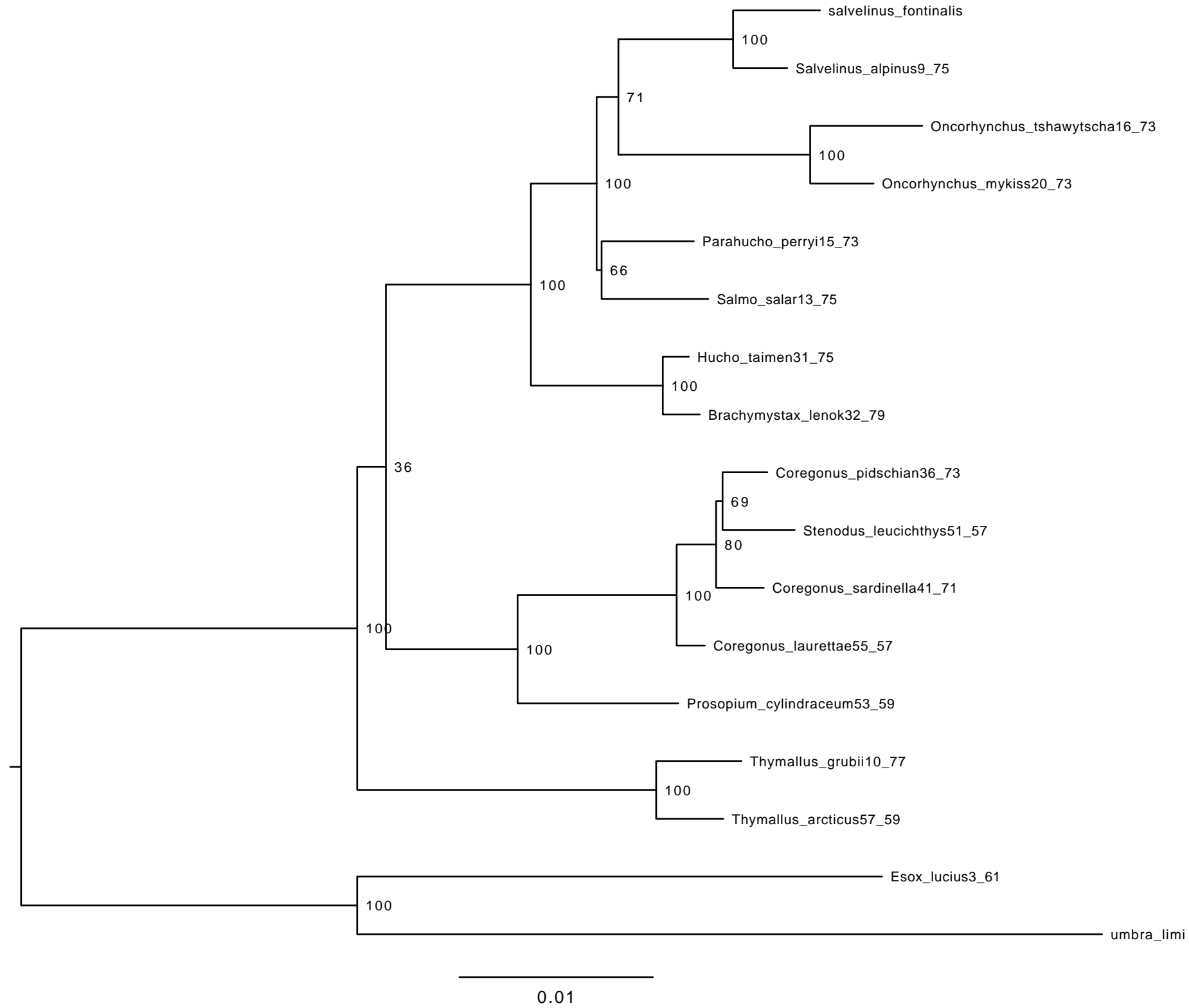

Supplement: Supplemental Information 1 — Assembled contigs matching UCE loci, alignments, raw code and tree files. [file peerj-08-9389-s001.zip › Data Supplement/RAxML trees/95Unpartitioned.pdf]

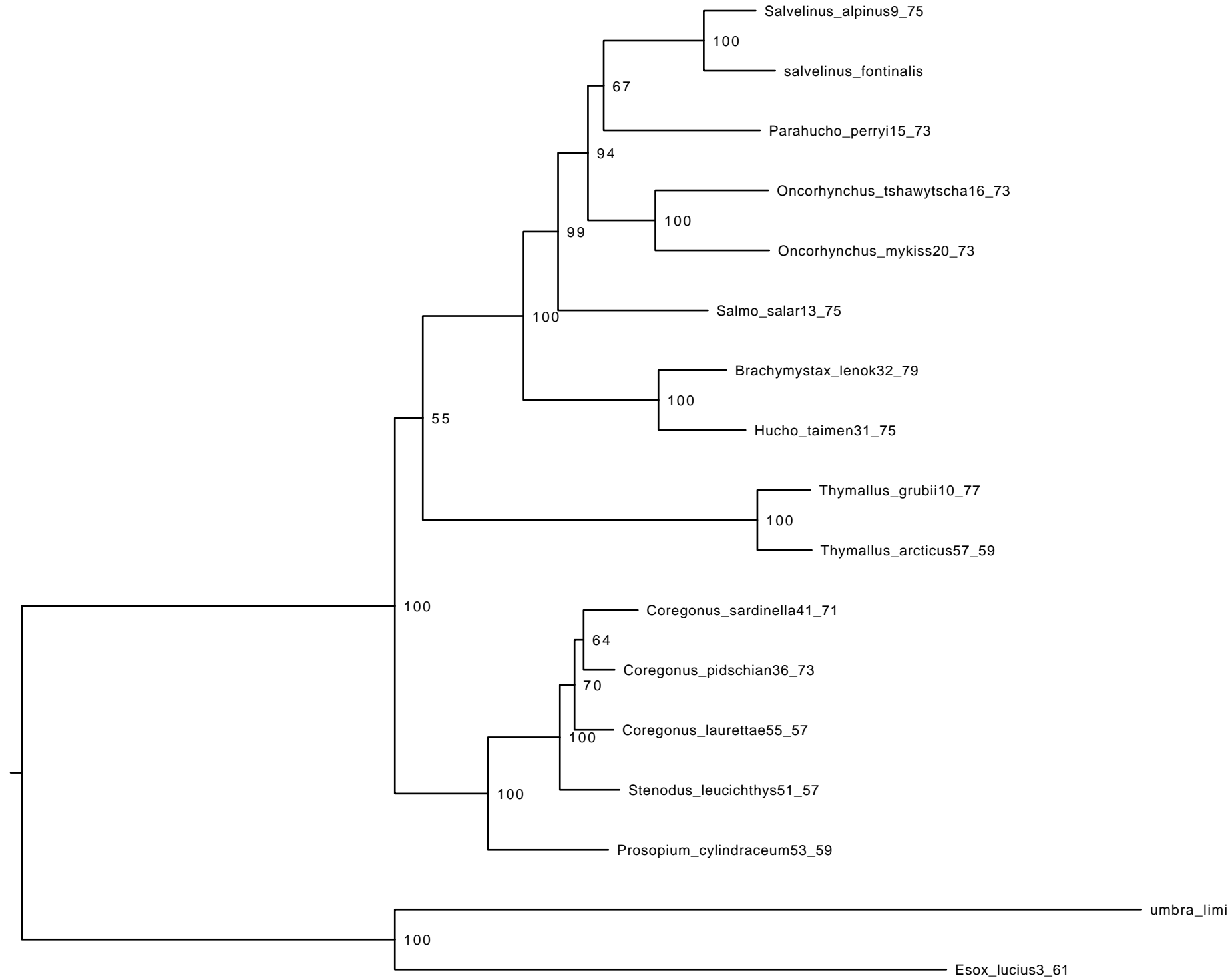

0.01

Supplement: Supplemental Information 1 — Assembled contigs matching UCE loci, alignments, raw code and tree files. [file peerj-08-9389-s001.zip › Data Supplement/RAxML trees/tetraByUCE.pdf]

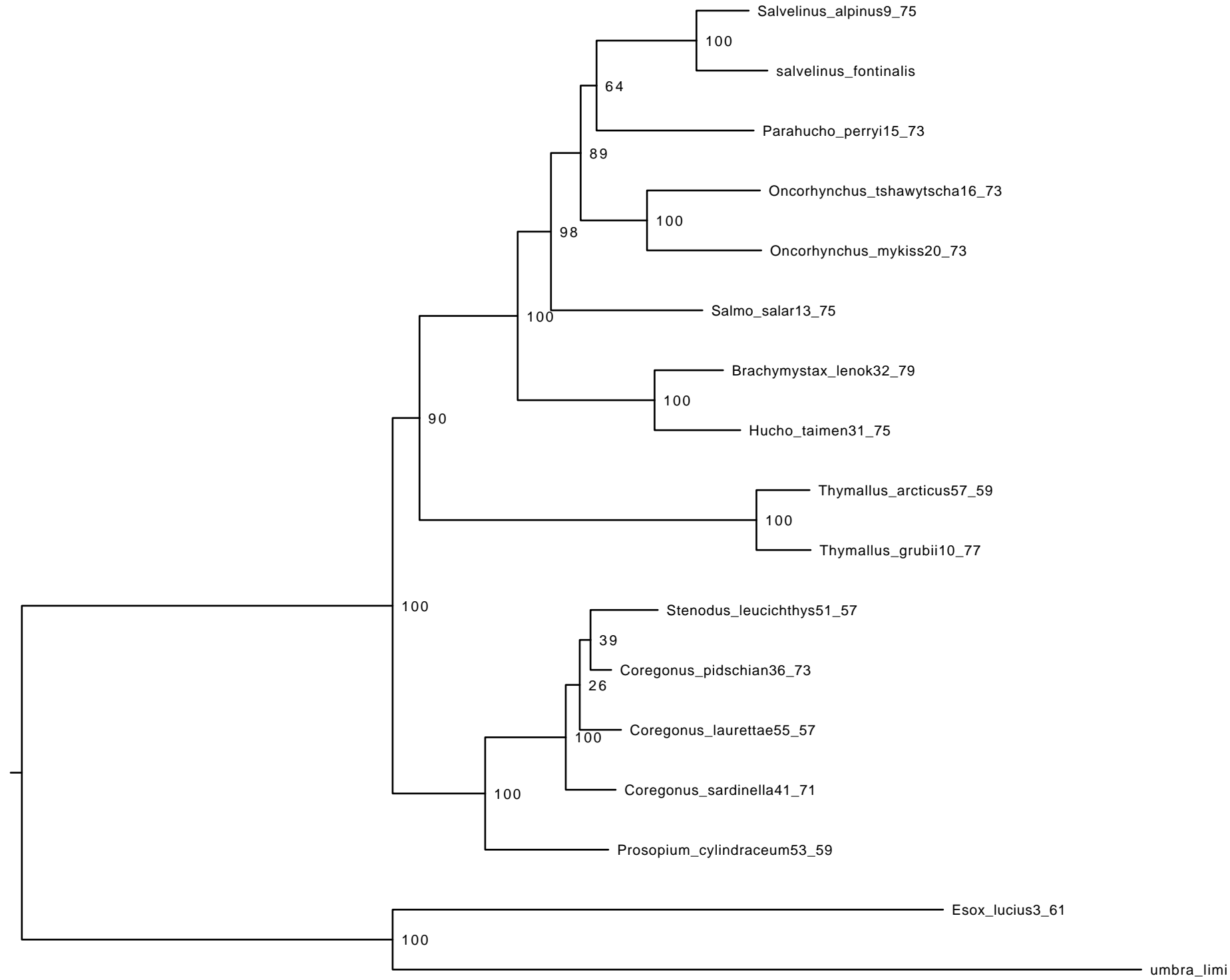

0.0090

Supplement: Supplemental Information 1 — Assembled contigs matching UCE loci, alignments, raw code and tree files. [file peerj-08-9389-s001.zip › Data Supplement/RAxML trees/tetraGreedy.pdf]

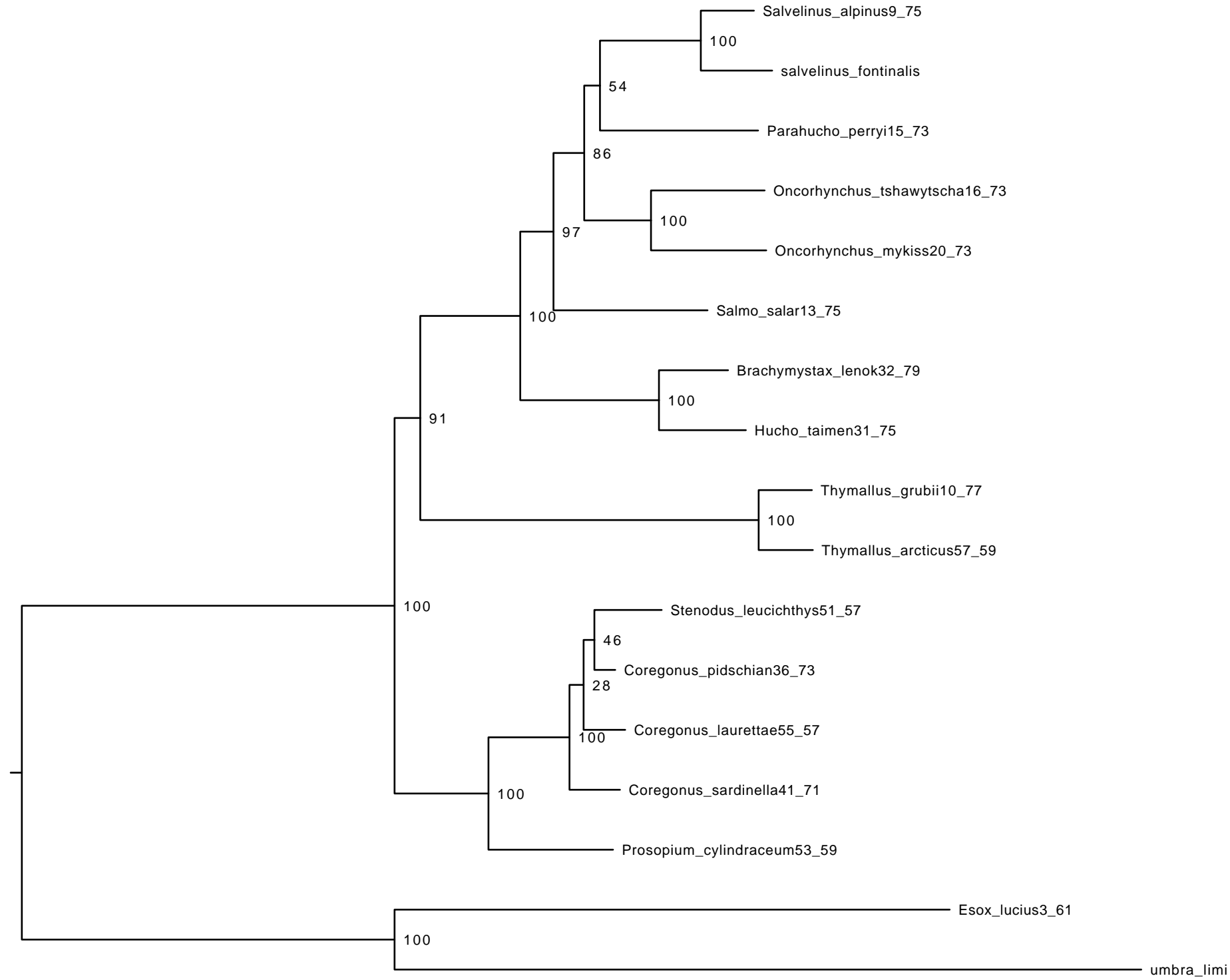

0.0090

Supplement: Supplemental Information 1 — Assembled contigs matching UCE loci, alignments, raw code and tree files. [file peerj-08-9389-s001.zip › Data Supplement/RAxML trees/tetraUnpartitioned.pdf]

# Animals

## Vertebrates

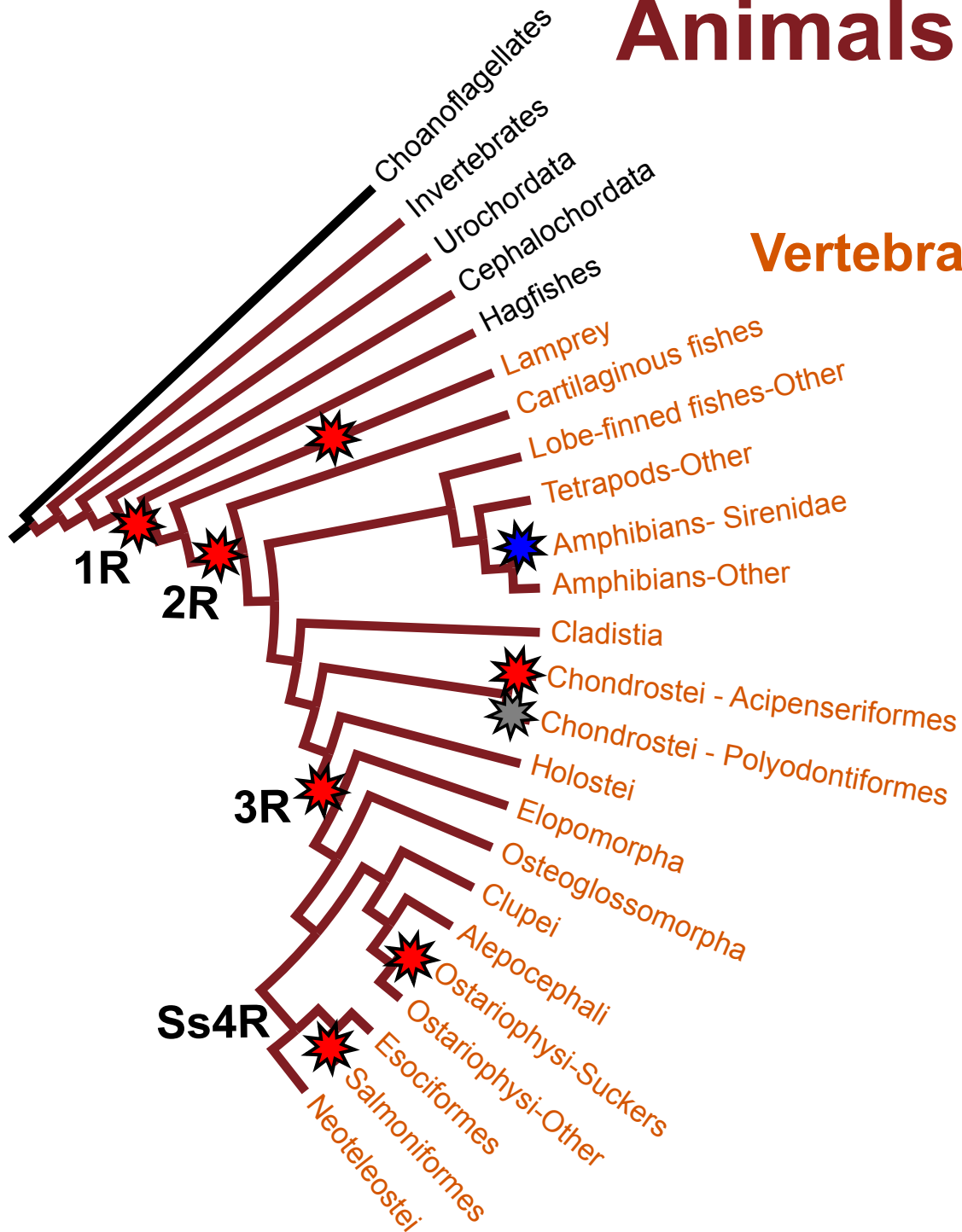

Supplement: Supplemental Information 2 — Ancient whole-genome duplications characteristic of all vertebrates (1R and 2R) are labeled along with the teleost-specific WGD (3R) and the salmonid-specific fourth round (Ss4R) whole-genome duplication. Modified from Campbell et al. (2016). [file peerj-08-9389-s002.pdf]
